# Synergistic Antifungal Activity of Synthetic Peptides and Antifungal Drugs against *Candida albicans* and *C. parapsilosis* Biofilms

**DOI:** 10.3390/antibiotics11050553

**Published:** 2022-04-21

**Authors:** Leandro P. Bezerra, Cleverson D. T. Freitas, Ayrles F. B. Silva, Jackson L. Amaral, Nilton A. S. Neto, Rafael G. G. Silva, Aura L. C. Parra, Gustavo H. Goldman, Jose T. A. Oliveira, Felipe P. Mesquita, Pedro F. N. Souza

**Affiliations:** 1 Department of Biochemistry and Molecular Biology, Federal University of Ceará, Fortaleza 60451, CE, Brazil; leandro.bioquimica@gmail.com (L.P.B.); ayrlesbrandao@gmail.com (A.F.B.S.); jacksoncesarc@gmail.com (J.L.A.); niltonararipeneto@hotmail.com (N.A.S.N.); alchaconp@unal.edu.co (A.L.C.P.); jtaolive@ufc.br (J.T.A.O.); 2 Department of Biology, Federal University of Ceará, Fortaleza 60451, CE, Brazil; rafaelguimaraes@ufc.br; 3 Faculty of Pharmaceutical Sciences of Ribeirão Preto, University of São Paulo, São Paulo P.O. Box 05508-000, SP, Brazil; ggoldman@usp.br; 4 Drug Research and Development Center, Department of Physiology and Pharmacology, Federal University of Ceará, Rua Coronel, Nunes de Melo 100, Caixa, Fortaleza 60430-275, CE, Brazil; felipe_mesquita05@hotmail.com

**Keywords:** antibiofilm activity, candidiasis, synergism, synthetic peptides, antifungal drugs

## Abstract

*C. albicans* and *C. parapsilosis* are biofilm-forming yeasts responsible for bloodstream infections that can cause death. Synthetic antimicrobial peptides (SAMPs) are considered to be new weapons to combat these infections, alone or combined with drugs. Here, two SAMPs, called *Mo*-CBP_3_-PepI and *Mo*-CBP_3_-PepIII, were tested alone or combined with nystatin (NYS) and itraconazole (ITR) against *C. albicans* and *C. parapsilosis* biofilms. Furthermore, the mechanism of antibiofilm activity was evaluated by fluorescence and scanning electron microscopies. When combined with SAMPs, the results revealed a 2- to 4-fold improvement of NYS and ITR antibiofilm activity. Microscopic analyses showed cell membrane and wall damage and ROS overproduction, which caused leakage of internal content and cell death. Taken together, these results suggest the potential of *Mo*-CBP_3_-PepI and *Mo*-CBP_3_-PepIII as new drugs and adjuvants to increase the activity of conventional drugs for the treatment of clinical infections caused by *C. albicans* and *C. parapsilosis*.

## 1. Introduction

Biofilms are established by microbial cells on an inert or living surface, promoting the development of microcolonies with polymeric matrices and enhancing the resistance to various antimicrobial agents [1,2]. *Candida* species are biofilm-forming yeasts responsible for up to 15% of hospital-acquired cases of sepsis [3]. A mature biofilm produced by *Candida* spp. consists of an extracellular matrix composed of glycoproteins (55%), carbohydrates (25%), lipids (15%), and nucleic acids (5%) [4]. The National Institute of Health (NIH) in the USA considers biofilms to be a public health problem and estimated that they can be responsible for 80% of the difficulties in curing human infections [1,2,4,5]. The most susceptible people are immunocompromised patients, AIDS+ patients, patients under chemotherapy treatment or immunosuppressive therapies, and patients fitted with medical devices (catheters, pacemakers, and heart valves) [6,7].

*C. albicans* and *C. parapsilosis* are common opportunistic fungal pathogens that asymptomatically colonize the mucosal surfaces and skin of healthy individuals. However, in some circumstances they can cause an infection called candidiasis [8]. In addition, *C. albicans* and *C. parapsilosis* are responsible for bloodstream infections termed candidemia, which are common in immunocompromised patients, including those in intensive care units [9]. Currently, the treatment of infections caused by *C. albicans* and *C. parapsilosis* involves the use of antifungal agents that interrupt different metabolic pathways of the cell. However, some studies have reported *Candida* resistance to these antifungal molecules [10,11,12]. A study by Katiyar and collaborators [13] described *Candida* clinical isolates that contain genes responsible for resistance to some commercial antifungal agents [13]. To counter this problem, synthetic antimicrobial peptides (SAMPs) have been described as new alternatives, either alone or combined with commercial antifungal drugs, to control *Candida* infection and overcome the pathogens’ resistance [1]. SAMPs have some important antimicrobial characteristics found in natural antimicrobial peptides, such as positive net charge, α-helical structure, low molecular weight (600–1200 Da), high hydrophobic ratio (40–60%) and amphipathicity [1,14]. 

Recently, our research group designed, characterized, and evaluated the antimicrobial activity of two synthetic peptides, called *Mo*-CBP_3_-PepI (CPIAQRCC) and *Mo*-CBP_3_-PepIII (AIQRCC). These peptides were designed based on the structure of *Mo*-CBP_3_, a chitin-binding protein purified from *Moringa oleifera* seeds [15,16]. The anticandidal activity and mechanism of action of these peptides were evaluated by Oliveira et al. (2019) and Lima et al. (2020). Therefore, the aim of this study was to evaluate the antifungal activity and action mechanism of *Mo*-CBP_3_-PepI and *Mo*-CBP_3_-PepIII, alone or combined with NYS and ITR, against *C. albicans* and *C. parapsilosis* biofilms.

## 2. Results

### 2.1. Antibiofilm Activity of Synthetic Peptides and Two Commercial Drugs 

**Figure 1:**
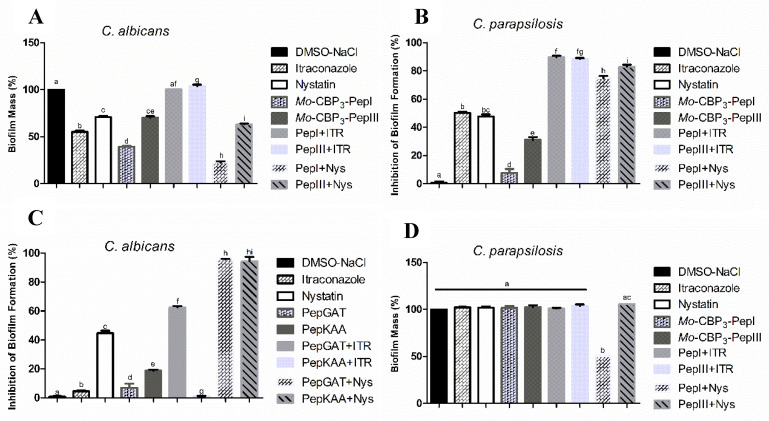
(**A**,**B**) Inhibitory activity of biofilm formation and (**C**,**D**) degradation of mature biofilm of *C. albicans* and *C. parapsilosis*. DMSO-NaCl was used as a negative control and ITR and NYS as positive controls. The letters represent the mean ± standard deviation of three replicates. Different lowercase letters indicate a statistically significant difference compared to DMSO-NaCl by analysis of variance (*p* < 0.05).

The activities of *Mo*-CBP_3_-PepI and *Mo*-CBP_3_-PepIII (50 μg mL^−1^) against *C. albicans* and *C. parapsilosis* biofilms are shown in Figure 1. The biofilm formation of *C. albicans* was inhibited 10% by *Mo*-CBP_3_-PepI, whereas *Mo*-CBP_3_-PepIII did not show any activity. Interestingly, the commercial drugs ITR and NYS inhibited biofilm formation by only 7% and 40%, respectively (Figure 1A). Regarding the synergistic effect, the combination of both peptides *Mo*-CBP_3_-PepI and *Mo*-CBP_3_-PepIII with ITR or NYS significantly enhanced the inhibition of *C. albicans* biofilm formation. For instance, the two peptides combined with NYS increased the inhibition of *C. albicans* biofilm formation by 40% to 80% (Figure 1A). ITR and NYS inhibited *C. parapsilosis* biofilm formation by 45% and 43%, respectively. In contrast, *Mo*-CBP_3_-PepI and *Mo*-CBP_3_-PepIII inhibited this by only 15% and 25%, respectively (Figure 1B). On the other hand, combinations of *Mo*-CBP_3_-PepI + ITR, *Mo*-CBP_3_-PepIII + ITR, *Mo*-CBP_3_-PepI + NYS, and *Mo*-CBP_3_-PepIII + NYS inhibited the biofilm formation by about 98%, 96%, 79%, and 82%, respectively (Figure 1B). 

Regarding the degradation of mature *C. albicans* biofilm, ITR and NYS decreased the biofilm mass by about 50% and 30%, while *Mo*-CBP_3_-PepI and *Mo*-CBP_3_-PepIII only degrading it by 60% and 30%, respectively (Figure 1C). Remarkably, the combinations *Mo*-CBP_3_-PepI + ITR and *Mo*-CBP_3_-PepIII + ITR did not have any effect (Figure 1C). However, the combinations *Mo*-CBP_3_-PepI + NYS and *Mo*-CBP_3_-PepIII + NYS degraded 85% and 50% of the mature *C. albicans* biofilm (Figure 1C). Concerning the degradation of mature *C. parapsilosis* biofilm, only the combination Mo-CBP3-PepI + NYS showed activity, reducing the biofilm biomass by 50% (Figure 1D).

### 2.2. Analysis of Candida Biofilm Morphology 

**Figure 2:**
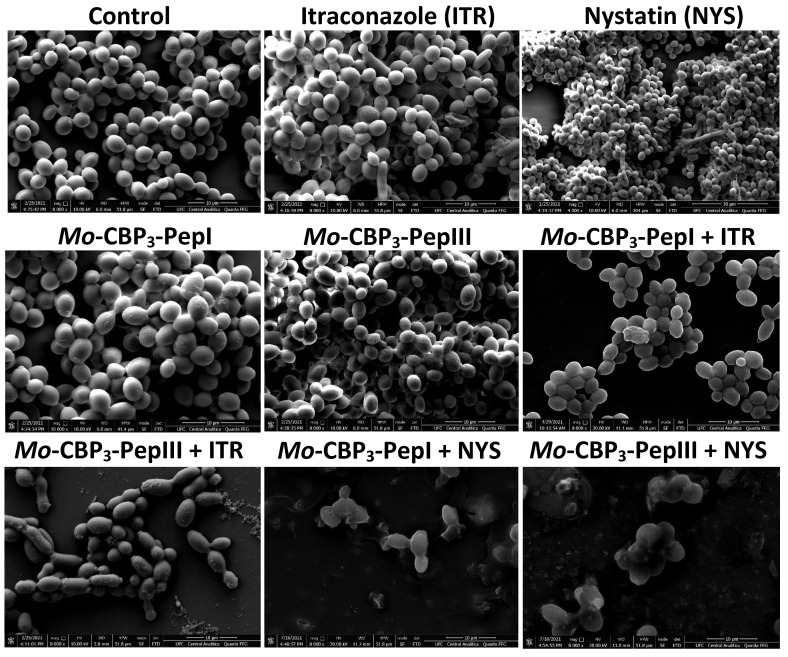
SEM images showing *C. albicans* biofilms after treatment with ITR, NYS, *Mo*-CBP_3_-PepI, *Mo*-CBP_3_-PepIII, and their combinations. Control: DMSO-NaCl solution.

**Figure 3:**
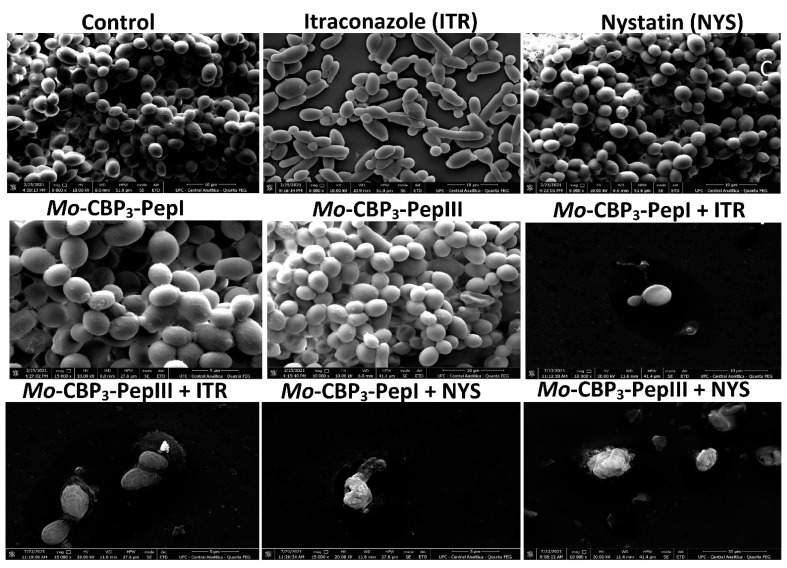
SEM images showing *C. parapsilosis* biofilms after treatment with ITR, NYS, *Mo*-CBP_3_-PepI, *Mo*-CBP_3_-PepIII, and their combinations. Control: DMSO-NaCl solution.

Scanning electron microscopy (SEM) was used to evaluate damage to *C. albicans* and *C. parapsilosis* biofilms after all treatments (Figure 2 and Figure 3). The control cells did not show any damage or alterations on the surface; only spherical-shaped cells were observed without cracks or scars. The treatment with peptides or drugs caused only mild damage, such as wrinkles and slight changes to the morphology of cells, which had a very similar appearance to the control (Figure 2 and Figure 3). In contrast, the combination of both peptides with the two drugs caused a significant reduction in the mature biofilm compared to control, and it was possible to see damage such as small blebs, new buds, scars, and rings of truncated bud scars. *Mo*-CBP_3_-PepI + NYS and *Mo*-CBP_3_-PepIII + NYS were by far the most lethal to *C. albicans* and *C. parapsilosis*. In those treatments, the cells were greatly damaged, with high roughness levels, severe alterations in morphology, and a clear indication of cell lysis leading to loss of cytoplasm (Figure 2 and Figure 3). 

**Figure 4:**
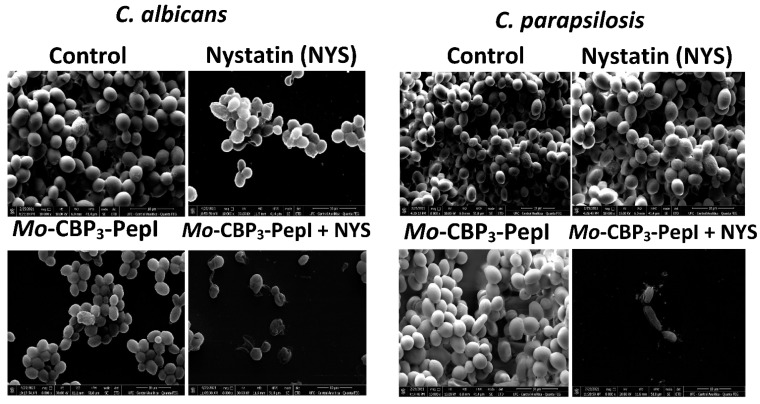
SEM images showing alterations of mature biofilm of *C. albicans* and *C. parapsilosis* after treatment with *Mo*-CBP_3_-PepI, NYS and *Mo*-CBP_3_-PepI + NYS. Control: DMSO-NaCl solution.

Because *Mo*-CBP_3_-PepI + NYS showed the best inhibitory activity against biofilm formation, this sample was chosen to investigate alteration of mature biofilms of *C. albicans* and *C. parapsilosis* (Figure 4). The control biofilm (treated with DMSO-NaCl) did not present any damage, while the biofilms treated with NYS or *Mo*-CBP_3_-PepI presented mild damage, such as altered morphology and wrinkles, distortion, and apparent reduction in biomass compared to the controls. However, *Mo*-CBP_3_-PepI + NYS was highly lethal to mature *C. albicans* and *C. parapsilosis* biofilms (Figure 4). These biofilms had a large reduction in biomass, as well as severe cell damage, such as depression-like cavities and damage to the cell wall, alterations in cell shape, wrinkles and scars, and loss of internal content (Figure 4).

### 2.3. Membrane Pore Formation

**Figure 5:**
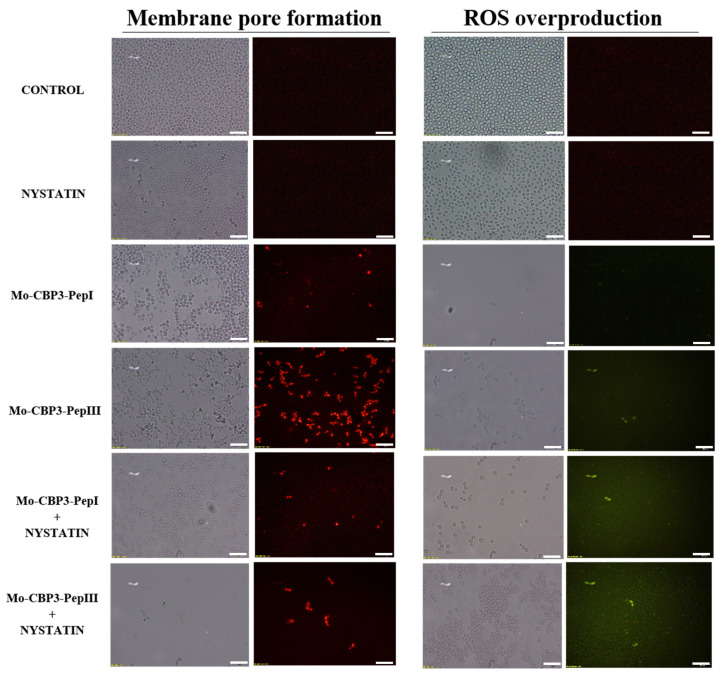
Fluorescence images showing membrane pore formation and ROS overproduction on inhibition of biofilm of *C. albicans* cells. Control solution of DMSO-NaCl, treated with *Mo*-CBP_3_-PepI and *Mo*-CBP_3_-PepIII at 50 μg mL^−1^ and synergistic activity of both peptides with NYS. Membrane pore formation was measured by the propidium iodide (PI) uptake assay, and ROS overproduction was detected using 2′, 7′ dichlorofluorescein diacetate (DCFH-DA). Bars: 100 µm.

**Figure 6:**
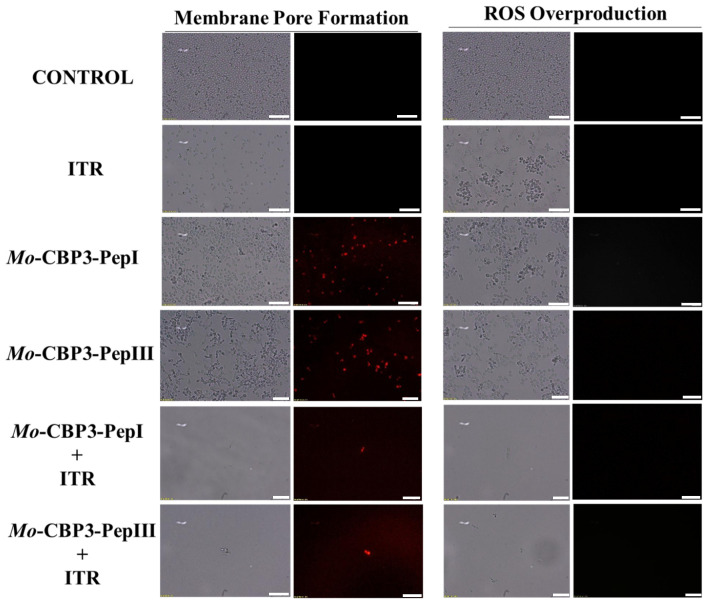
Fluorescence images showing membrane pore formation and ROS overproduction on inhibition of the biofilm of *C. parapsilosis* cells. Control solution of DMSO-NaCl, treated with *Mo*-CBP_3_-PepI and *Mo*-CBP_3_-PepIII at 50 μg mL^−1^ and synergistic activity of both peptides with ITR. Membrane pore formation was measured by the PI uptake assay, and ROS overproduction was detected using 2′, 7′ dichlorofluorescein diacetate (DCFH-DA). Bars: 100 µm.

**Figure 7:**
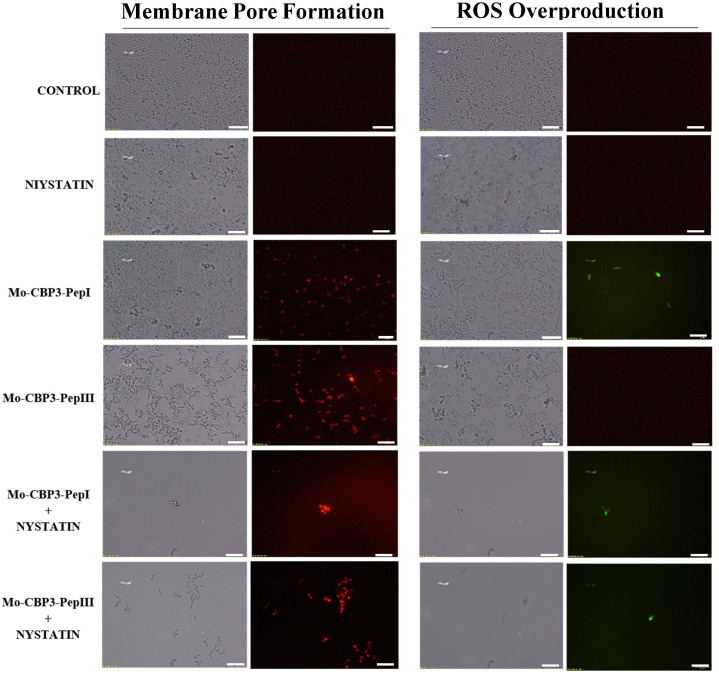
Fluorescence images showing membrane pore formation and ROS overproduction on inhibition of the biofilm of *C. parapsilosis* cells. Control solution of DMSO-NaCl, treated with *Mo*-CBP3-PepI and Mo-CBP3-PepIII at 50 μg mL^−1^ and synergistic activity of both peptides with NYS. Membrane pore formation was measured by the PI uptake assay, and ROS overproduction was detected using 2′, 7′ dichlorofluorescein diacetate (DCFH-DA). Bars: 100 µm.

**Figure 8:**
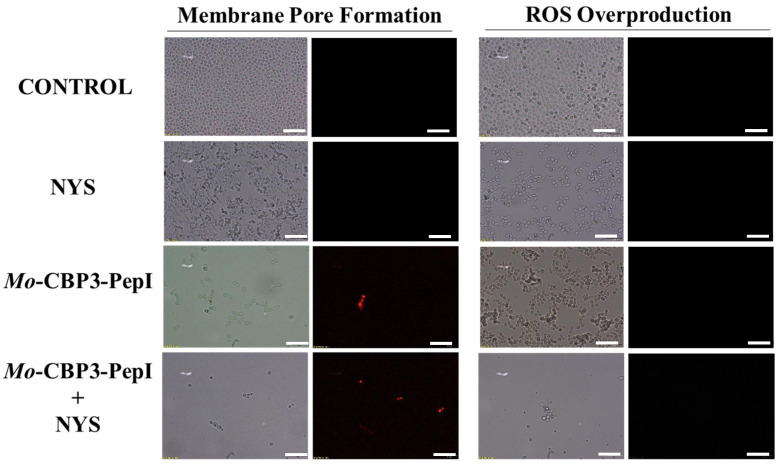
Fluorescence images showing membrane pore formation and ROS overproduction on degradation of the biofilm of *C. albicans* cells. Control solution of DMSO-NaCl, treated with *Mo*-CBP3-PepI at 50 μg mL^−1^ and synergistic activity with NYS. Membrane pore formation was measured by the PI uptake assay, and ROS overproduction was detected using 2′, 7′ dichlorofluorescein diacetate (DCFH-DA). Bars: 100 µm.

**Figure 9:**
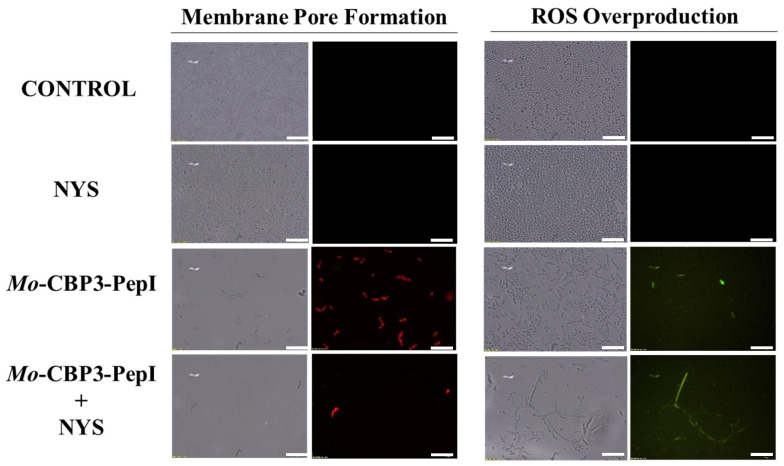
Fluorescence images showing membrane pore formation and ROS overproduction on degradation of the biofilm of *C. parapsilosis* cells. Control solution of DMSO-NaCl, treated with *Mo*-CBP_3_-PepI at 50 μg mL^−1^ and synergistic activity with NYS. Membrane pore formation was measured by the PI uptake assay, and ROS overproduction was detected using 2′, 7′ dichlorofluorescein diacetate (DCFH-DA). Bars: 100 µm.

The propidium iodide (PI) uptake assay was used to evaluate possible damage to the yeast cell membranes. PI interacts with DNA, releasing red fluorescence, but this is only possible when the membrane is damaged, since healthy membranes are impermeable to PI. As expected, the control (DMSO-NaCl solution) did not damage the cell membranes, because no fluorescence was detected. Similarly, cells treated with NYS and ITR did not show any fluorescence. However, *Mo*-CBP_3_-PepI and *Mo*-CBP_3_-PepIII, alone or in combination with NYS or ITR, induced red fluorescence in *C. albicans* and *C. parapsilosis* cells, indicating these cells membranes were damaged (Figure 5, Figure 6, Figure 7, Figure 8 and Figure 9).

ROS overproduction is another mechanism employed by peptides to inhibit biofilm formation. The results showed that treatment of *C. albicans* cells with NYS or ITR did not induce ROS overproduction, whereas both peptides and their combination with NYS induced a slight production of ROS. None of the treatments induced ROS overproduction by *C. parapsilosis* biofilms (Figure 5, Figure 6, Figure 7, Figure 8 and Figure 9).

### 2.4. Molecular Docking

**Figure 10:**
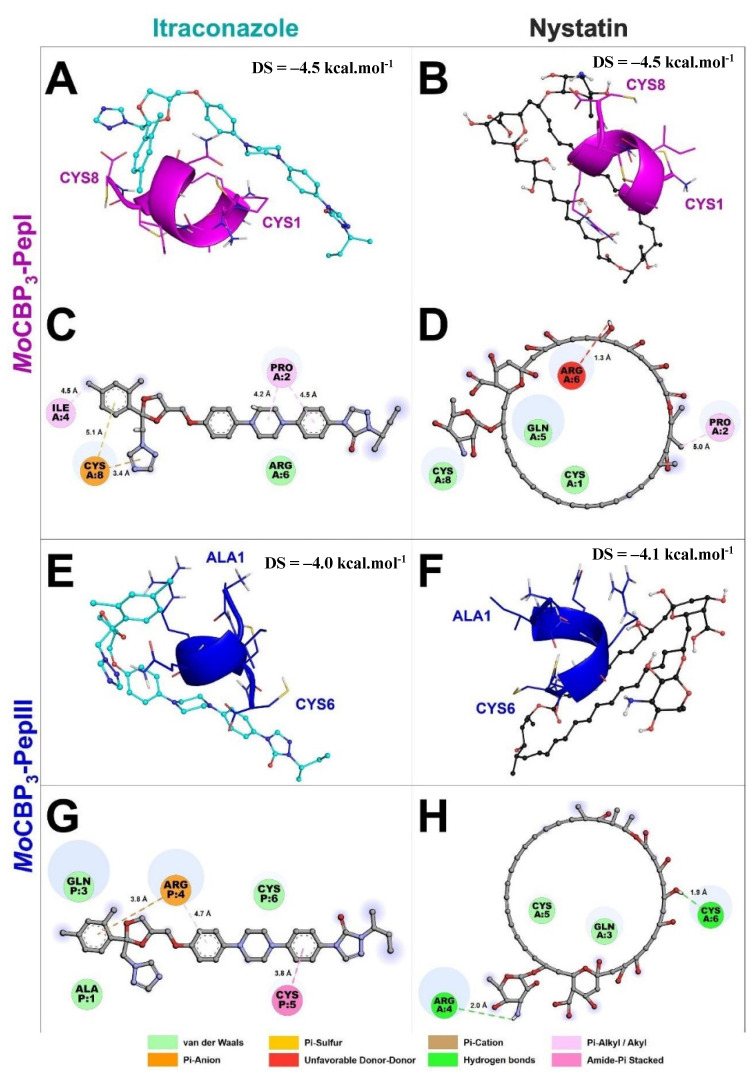
Molecular docking revealed that *Mo*-CBP_3_-PepI and *Mo*-CBP_3_-PepIII interact with ITR and NYS. *Mo*-CBP_3_-PepI is represented in pink (**A**,**B**) and *Mo*-CBP_3_-PepIII in blue (**E**,**F**). (**C**,**D**,**G**,**H**) show the binding sites of *Mo*-CBP_3_-PepI and *Mo*-CBP_3_-PepIII with ITR and NYS.

The molecular docking assays were performed to evaluate the possible interactions of the peptides with NYS and ITR. *Mo*-CBP_3_-PepI interacted with ITR and NYS with binding interaction energy (LBIE) values of −4.5 and −4.2 kcal.mol^−1^, respectively (Figure 10A,B). The amino acid residues Pro2 and Ile4 of the Mo-CBP3-PepI peptide showed Pi-Alkyl interactions with the phenyl (4.5 Å), piperazine (4.2 Å), and dichlorophenyl (4.5 Å) groups of ITR. Cys8 had a Pi-Anion (3.4 Å, triazole group) and a Pi-Sulfur (5.1 Å, dichlorophenyl group) interaction with ITR, and Arg6 presented only van der Waals interaction (Figure 10C). *Mo*-CBP_3_-PepI interacted with NYS by van der Waals forces between Cys8, Gln5, and Cys1. An Alkyl (5.0 Å) interaction with Pro2 and an unfavorable donor-donor (1.3 Å) with Arg6 were also observed (Figure 10D).

*Mo*-CBP_3_-PepIII presented docking scores of −4.0 and −4.1 kcal.mol^−1^ with ITR and NYS, respectively (Figure 10E,F). *Mo*-CBP_3_-PepIII interacted through van der Waals forces through residues Ala1, Gln3, and Cys6 with ITR. Cys5 interacted through an Amide-Pi stacked (3.8 Å) with the phenyl group of ITR. The Arg4 of *Mo*-CBP_3_-PepIII established a Pi-Cation interaction with the dichlorophenyl group (3.8 Å) and a Pi-Alkyl interaction (4.7 Å) with the methoxyphenyl group of itraconazole (Figure 10E,G). The interaction between *Mo*-CBP_3_-PepIII and NYS was supported by hydrogen bonds between residues Arg4 (2.0 Å) and Cys6 (1.9 Å), as well as through van der Waals interactions through residues Gln3 and Cys5 (Figure 10F,H).

### 2.5. Hemolytic Assay

As shown in a previous study [16], *Mo*-CBP_3_-PepI and *Mo*-CBP_3_-PepIII had no hemolytic activity against any human blood type tested (Table 1), even at 50 μg mL^−1^. In contrast, NYS (1000 μg mL^−1^) caused 100% hemolysis in all human blood types, and ITR (1000 μg mL^−1^) caused 75%, 68%, and 58% hemolysis to Type A, B, and O red blood cells, respectively (Table 1). 

In general, the combination of synthetic peptides decreased the hemolytic effect of both drugs (Table 1). The combination of *Mo*-CBP_3_-PepI with NYS resulted in hemolytic effects of 14%, 23%, and 2%, and the combination of *Mo*-CBP_3_-PepI with ITR resulted in 0%, 4%, and 8% hemolysis to Type A, B, and O red blood cells, respectively (Table 1). The combination of *Mo*-CBP_3_-PepIII with NYS hemolyzed 45%, 30%, and 18%, while the combination of *Mo*-CBP_3_-PepI with ITR resulted in 50%, 15%, and 2% for Type A, B, and O red blood cells, respectively (Table 1).

## 3. Discussion

Natural antimicrobial peptides (AMPs) are promising molecules to act as substitutes or adjuvants to treat infections. However, they have some disadvantages, such as toxicity, low resistance to proteolysis, and the high cost of isolation and purification. The development of synthetic antimicrobial peptides (SAMPs) is an alternative solution to overcome these drawbacks, since they have low or no toxicity to mammalian cells, and low chance of developing antimicrobial resistance based on their mechanism of action [1,14]. 

Bioinspired SAMPs based on natural AMPs can have attributes that are not present in the natural molecule [17,18]. A good example is the synthetic peptide LAH4, designed based on the Magainin 2 sequence, which presented potent activity against *Escherichia coli* and *Staphylococcus aureus* compared with the natural peptide Magainin 2 [17,18]. Recently, our research group designed peptides derived from *Mo*-CBP_3_ and antifungal chitin-binding protein from *M. oleifera* seeds. *Mo*-CBP_3_-PepI and *Mo*-CBP_3_-pepIII inhibited the growth of *C. albicans* and *C. parapsilosis* planktonic cells by the stimulation of ROS production, cell wall damage, and membrane pore formation, leading to death [15,16]. It is important to mention that *Mo*-CBP_3_ did not present anticandidal activity. Based on that, we decided to evaluate the potential of *Mo*-CBP_3_-PepI and *Mo*-CBP_3_-pepIII to inhibit biofilm formation and its capacity to promote degradation of mature biofilms of *C. albicans* and *C. parapsilosis*.

Regarding degradation of the mature biofilms of *C. albicans*, *Mo*-CBP_3_-PepI and *Mo*-CBP_3_-PepIII had activity of 40% and 70%, respectively (Figure 1). These results corroborate those involving gH625, a peptide analog from gH625-M, which reduced by 61% the biomass of mature biofilms of *C. albicans* [19]. SEM analysis of *C. albicans* and *C. parapsilosis* treated with *Mo*-CBP_3_-PepI and *Mo*-CBP_3_-PepIII showed that the biofilms suffered severe structural damage. Furthermore, SEM images suggested that the two peptides induced rupture of the cell wall and membrane pore formation, leading to internal content loss and death. The images also showed the presence of scars, buds, and cracks. These results corroborate those reported by Belmadani and collaborators [20], who observed that Dermaseptin-S1, an antimicrobial peptide from *Phyllomedusa sauvagii*, decreased *C. albicans* biofilm formation by causing changes in the cell wall structure, membrane pore formation, and leakage of internal content. Similar behavior was observed by Sierra et al., where a *C. albicans* biofilm suffered severe damage by the antimicrobial peptide called Histatin-5 [21]. This severe damage observed in the cell wall of both cells via SEM analysis can be explained since *Mo*-CBP_3_-PepI and *Mo*-CBP_3_-PepIII are designed based on the sequence of *Mo*-CBP_3_, which is a chitin-binding protein from *M. oleifera* seeds [16]. Both peptides can interact with the chitin present in the fungal cell wall and cause destabilization of the cell, leading to rupture, electrolyte imbalance, and thus cell death.

Unlike many commercial drugs that have specific targets, SAMPs target the cell membrane and/or the cell wall [14]. The ability of SAMPs to alter the microbial membrane permeability is considered the most common mechanism of action of these molecules, making the development of resistance mechanisms by microorganisms very difficult [1,14]. Fluorescence microscopy analyses were performed to evaluate if our peptides could induce membrane damage. *Mo*-CBP_3_-PepI and *Mo*-CBP_3_-PepIII induced PI uptake in *C. albicans* and *C. parapsilosis* biofilms, suggesting pore formation or cell membrane damage, as observed by SEM analysis. Furthermore, the peptides induced ROS overproduction in *C. parapsilosis* and *C. albicans* biofilms. A similar profile was observed using the peptides KP and MCh-AMP1, which are synthetic peptides able to induce ROS overproduction in *C. albicans* biofilm, leading to cell death. ROS are involved in the damage of essential molecules such as proteins, lipids, and DNA [22].

There are some explanations for the synergistic effect of the peptides and antifungal drugs tested here. First, the interactions between both peptides and NYS and ITR (Figure 6) can explain the synergistic activity obtained, where both peptides enhanced the activity of both drugs. Additionally, molecular docking studies were performed to evaluate whether both peptides could interact with NYS and ITR. Similar behavior was detected by Souza et al. [23], where *Mo*-CBP_3_-PepI and *Mo*-CBP_3_-PepIII interacted with griseofulvin by weak interactions, such as hydrogen bonds and hydrophobic interactions. The interaction of peptides with griseofulvin enhanced its activity against dermatophytes and reduced the toxicity of the drug, as was also shown in this study.

Two hypotheses can could explain the synergistic action between peptides and NYS. First, peptides target membranes and NYS targets the ergosterol. The interaction between peptides and NYS could result in a coordinated attack on the *Candida* membrane, enhancing the deleterious effect on it. Second, besides targeting the ergosterol in the membrane, NYS also has intracellular targets [24]. Once within the cytoplasm, NYS can attack the vacuole, causing its enlargement and impairing its function. Due to membrane-pore formation, the peptides might facilitate the access of NYS to the cytoplasm. It is known that *Mo*-CBP_3_-PepI and *Mo*-CBP_3_-PepIII form pores of 6 and 20 kDa, respectively, in *C. albicans* and *C. parapsilosis* membranes [15,16]. NYS has a molecular weight of 926.1 Da, so it is feasible to suggest that NYS passes through the membrane and attacks the cellular vacuole.

The synergistic effect of peptides with ITR, which has a molecular weight of 705 Da, could be explained by its the passage through the membrane by the pores formed in it as a result of the peptides’ action. The facilitated passage of ITR through the pores formed by peptides in the membrane enhances its activity of inhibiting the cholesterol biosynthesis pathways, and thus the ergosterol synthesis [25]. One relevant fact is that both *Mo*-CBP_3_-PepI and *Mo*-CBP_3_-PepIII improved the activity of NYS and ITR by up to 50% regarding the inhibition of biofilm formation of *C. albicans* and *C. parapsilosis*. Moreover, the results showed that *Mo*-CBP_3_-PepI enhanced NYS activity up to 60% in degrading the mature biofilms and preformed biofilm of both yeasts. The peptides also enhanced the antifungal activity of NYS and ITR against *C. albicans* and *C. parapsilosis* biofilms.

ITR and NYS can cause undesired effects, such as vomiting, nausea, diarrhea, anorexia, abdominal pain, and dizziness. Besides these side effects, cardiotoxicity and hypertension have been attributed to ITR usage. An unexpected and interesting result was that the association of peptides with antifungal drugs reduced their toxicity to human erythrocytes. For example, NYS alone caused hemolysis of 100% in type-A erythrocytes, while *Mo*-CBP_3_-PepI + NYS and *Mo*-CBP_3_-PepIII + NYS induced hemolysis of 0 and 45%, respectively, for type A blood. All treatments combining peptides with antifungal drugs were able to reduce the drugs’ hemolytic effects.

Molecular docking analysis between peptides and drugs revealed a clue about how peptides reduced these hemolytic effects. The membrane of erythrocytes has neutral phospholipids, which means that any interaction with those membranes must be driven by hydrophobic interactions [24]. It is known that NYS and ITR are hydrophobic drugs [25,26]. Thus, hydrophobic interactions with membranes of erythrocytes may drive the hemolytic activity of NYS and ITR. The molecular docking experiments revealed that peptides had hydrophobic interactions with NYS and ITR, suggesting that the hydrophobic interactions between peptides and both drugs prevented the interaction with the erythrocyte membranes, reducing their hemolytic effect.

## 4. Materials and Methods

### 4.1. Ethics Statement

Does not apply to this study.

### 4.2. Biological and Chemical Materials

*C. albicans* (ATCC 10231) and *C. parapsilosis* (ATCC 22019) were obtained from the Laboratory of Plant Toxins of the Department of Biochemistry and Molecular Biology of Federal University of Ceará, Brazil. All chemicals were purchased from Sigma-Aldrich Co. (St. Louis, MO, USA).

### 4.3. Peptide Synthesis

The synthetic peptides *Mo*-CBP_3_-PepI (CPIAQRCC) and *Mo*-CBP_3_-PepIII (AIQRCC) were chemically synthesized by the company GenOne (São Paulo, Brazil), and the quality and purity (≥95%) were analyzed by reverse-phase high-performance liquid chromatography (RP-HPLC, Jasco, Easton, MD, USA) and mass spectrometry (Waltham, MA, USA).

### 4.4. Biological Activity

#### Antibiofilm Assay

The assays against *C. albicans* and *C. parapsilosis* biofilms were performed following the method described by [27,28,29], with some modifications. To evaluate the inhibition of the biofilm formation, 100 μL of *C. albicans* or *C. parapsilosis* suspension (2.5 × 10^3^ CFU/mL in Sabouraud liquid medium) was incubated in 96-well plates with 100 μL of *Mo*-CBP_3_-PepI, *Mo*-CBP_3_-PepII or *Mo*-CBP_3_-PepIII (50 µg mL^−1^, as defined by [14,15,16,23]), at 37 °C for 48 h. The supernatant was removed and the wells were washed three times with sterile 0.15 M NaCl. Next, the cells were fixed with 100 μL of 100% methanol for 15 min at 37 °C and the plates were air-dried under the same conditions. Then, 200 μL of an aqueous solution of 0.1% crystal violet was added and incubated for 30 min at 24 °C. To remove the excess crystal violet, the plates were washed three times with distilled water and finally 100 μL of 33% acetic acid to solubilize the dye bound in the biofilm. After 15 min, the absorbance was measured at 600 nm using an automated microplate reader (Epoch, Biotek, Santa Clara, CA, USA).

To evaluate the degradation of mature biofilm, the cell suspensions of both yeasts (100 μL, 2.5 × 10^3^ CFU/mL in Sabouraud liquid medium) were first incubated at 37 °C for 24 h in 96-well plates. Then, the supernatant was removed, and 100 μL of the Sabouraud liquid medium and 100 μL of each peptide (50 µg mL^−1^) were added and incubated again for 24 h. The culture medium was again discarded, and the same procedure that used 0.1% crystal violet was employed to quantify the biofilm mass. In both experiments, a solution of 5% DMSO in 0.9% NaCl was used as a negative control. NYS (1000 μg mL^−1^) and ITR (1000 μg mL^−1^) were used as positive controls. The synergism assays were carried out by combining the peptides (50 µg mL^−1^) with NYS or ITR (1000 µg mL^−1^) and the effectiveness was compared with the activity of the peptides and drugs alone.

### 4.5. Overproduction of Reactive Oxygen Species (ROS) 

The ROS overproduction was determined following the method described by Dias et al. [29], with some modifications. *C. albicans* and *C. parapsilosis* were incubated with the three peptides under the same conditions as described above. Then, 50 μL of cell suspension (2.5 × 10^3^ CFU/mL) was incubated with 50 μL of each peptide (50 µg mL^−1^) for 24 h and the formed biofilm was washed with 0.15 M NaCl three times to remove the Sabouraud liquid medium. Next, 20 μL of 2′,7′ dichlorofluorescein diacetate (DCFH-DA, Sigma, St. Louis, MI, USA) was added and incubated in the dark for 30 min at 24 °C. Finally, the biofilms were washed with 0.15 M NaCl and observed under a fluorescence microscope (Olympus System BX 41, Tokyo, Japan) with an excitation wavelength of 488 nm and emission wavelength of 525 nm.

### 4.6. Cell Membrane Integrity Assay

The cell membrane integrity of *C. albicans* and *C. parapsilosis* was tested as described by Dias et al. [29], with some modifications. The biofilms were treated as described for ROS overproduction analysis. Thus, 20 μL of propidium iodide (PI, Sigma, St. Louis, MI, USA) was added and incubated in the dark for 30 min at 24 °C. Then the samples were washed three times with 0.15 M NaCl to remove the excess of PI and observed with a fluorescence microscope (Olympus System BX 41, Tokyo, Japan) with an excitation wavelength of 535 nm and emission wavelength of 617 nm.

### 4.7. Scanning Electron Microscopy (SEM) Analysis

The morphological changes in the cells of *C. albicans* and *C. parapsilosis* were evaluated by SEM (Billerica, MA, USA), using the method described by Staniszewska et al. [30]. Biofilms were fixed with 1% (*v*/*v*) glutaraldehyde in 0.15 M sodium phosphate buffer at pH 7.0 for 16 h. Then the biofilms were washed with 0.15 M sodium phosphate buffer (pH 7.0) three times. Next, 0.2% (*v*/*v*) osmium tetroxide was added to the samples and incubated for 30 min at 37 °C and washed again under the same conditions described above. Samples were successively dehydrated with increased ethanol concentrations (30%, 50%, 70% 100% and 100% [*v*/*v*]) for 10 min each at 24 °C. Last, the final dehydration was realized with 50% hexamethyldisilane (HMDS, Sigma, St. Louis, MI, USA) diluted in ethanol for 10 min and then 100% HDMS. The biofilms were placed on stubs and coated with a 20 nm gold layer using a positron-emission tomography (PET) coating machine (Emitech-Q150TES, Quorum Technologies, Lewes, England). The images were obtained with an FEI inspectTM50 scanning electron microscope, equipped with a low energy detector (Everhart-Thornley), and the acceleration used was 20,000 kV and 20,000× detector magnification.

### 4.8. Obtainment, File Preparation, and Molecular Docking 

The three-dimensional (3D) structures of *Mo*-CBP_3_-PepI and *Mo*-CBP_3_-PepIII were predicted using the PepFold server 3 (https://bioserv.rpbs.univ-paris-diderot.fr/services/PEP-FOLD/ accessed on 15 February 2022) [31]. The amino acid protonation of the peptides was adjusted to pH 7.4 using Protein Prepare [32]. NYS (accession number CID 16219709) and ITR (accession number CID 55283) 3D structures were obtained from the database of PubChem (https://pubchem.ncbi.nlm.nih.gov/ accessed on 15 February 2022) [33]. The protonation of the ligands was adjusted using the Marvin Sketch software version 15.6.15. The energy minimization of the peptide hydrogens and the ligand was conducted using Discovery Studio v. 20.1 (https://discover.3ds.com/discovery-studio-visualizer-download accessed on 15 March 2022) and Open Babel version 2.4.0 (https://osdn.net/projects/sfnet_openbabel/downloads/openbabel/2.4.0/OpenBabel-2.4.0.exe/ accessed on 5 March 2022).

Molecular docking assays were carried out using Autodock Vina, version 1.1.2 [34]. Additionally, the Autodock graphical interface version 1.5.6 was used to maintain polar hydrogens and provide charges to peptides and drugs using Kollman united charges [35]. The *Mo*-CBP_3_-PepI and *Mo*-CBP_3_-PepIII were considered rigid molecules, and NYS and ITR were docked as flexible molecules. The grid box was defined as a 24 Å × 24 Å × 24 Å cube with the peptides in the center. The exhaustiveness was set to 16, and all other parameters were used as default. The software Discovery Studio v. 20.1 and the 3D interaction representations were realized using the Pymol program (https://pymol.org/2/ accessed on 8 March 2022).

### 4.9. Hemolytic Assay 

The hemolytic activities of *Mo*-CBP_3_-PepI, *Mo*-CBP_3_-PepIII, NYS, and ITR, alone and in their different combinations, were assessed using A, B, and O types of human erythrocytes as described by Souza et al. [14]. The concentrations of all solutions were the same as used in the synergism assays. The blood types were provided by the Hematology and Hemotherapy Center of Ceará (Fortaleza, Brazil).

The blood was collected in a tube with heparin (5 IU mL^−1^, Sigma Aldrich, São Paulo, Brazil), centrifuged at 300× *g* for 5 min at 4 °C, washed with sterile 0.15 M NaCl, and diluted to a concentration of 2.5%. Each blood type was incubated (100 µL) with solutions of Mo-CBP3-PepI, Mo-CBP3-PepIII (50 µg mL^−1^), NYS (1000 µg mL^−1^), or ITR (1000 µg mL^−1^) for 30 min at 37 °C, followed by centrifugation (300× *g* for 5 min at 4 °C, centrifuge Eppendorf 5810, Hannover, Germany). Supernatants were collected and transferred to 96-well microtiter plates and the hemolysis (%) was calculated by reading the absorbance at 414 nm using an automated absorbance microplate reader using DMSO-NaCl solution (0%) and 0.1% Triton X-100 (100%) as negative and positive controls for hemolysis, respectively. The hemolysis was calculated by the equation: [(Abs_414nm_ of sample treated with peptides or drugs-Abs_414nm_ of samples treated with DMSO-NaCl)/[(Abs_414nm_ of samples treated with 0.1% TritonX-100-Abs_414nm_ of samples treated with DMSO-NaCl] × 100.

### 4.10. Statistical Analysis 

All the assays were performed individually three times and the values are expressed as the mean ± standard error. The data were submitted to ANOVA followed by the Tukey test. GraphPad Prism version 5.01 (GraphPad Software company, Santa Clara, CA, USA) was used to generate all graphics, with a significance of *p* < 0.05.

## 5. Conclusions

The antibiofilm activity, absence of toxicity, and synergistic effect enhancing the activity of NYS and ITR, strongly indicated that *Mo*-CBP_3_-PepI and *Mo*-CBP_3_-PepIII are promising antibiofilm peptides which could act as new antimicrobial agents. We also highlight their use for clinical application or adjuvants to conventional drugs to overcome resistance developed by *Candida* species.

## Figures and Tables

**Table 1:** Hemolytic activity of *Mo*-CBP_3_-PepI and *Mo*-CBP_3_-PepIII, antifungal drugs, and combined solutions on human red blood cells.

Peptides/Combinations	% Hemolysis		
	Type A Blood	Type B Blood	Type O Blood
0.1% Triton X-100	100 ± 0.002	100 ± 0.001	100 ± 0.007
DMSO-NaCl Solution	0	0	0
NYS (1000 µg mL^−1^)	100 ± 0.005	100 ± 0.001	100 ± 0.002
ITR (1000 µg mL^−1^)	75 ± 0.007	68 ± 0.004	58 ± 0.003
*Mo*-CBP_3_-PepI (50 µg mL^−1^)	0	0	0
*Mo*-CBP_3_-PepIII (50 µg mL^−1^)	0	0	0
*Mo*-CBP_3_-PepI (50 µg mL^−1^) + NYS (1000 µg mL^−1^)	14 ± 0.006	23 ± 0.009	2 ± 0.001
*Mo*-CBP_3_-PepI (50 µg mL^−1^) + ITR (1000 µg mL^−1^)	0	4 ± 0.003	8 ± 0.005
*Mo*-CBP_3_-PepIII (50 µg mL^−1^) + NYS (1000 µg mL^−1^)	45 ± 0.001	30 ± 0.001	18 ± 0.007
*Mo*-CBP_3_-PepIII (50 µg mL^−1^) + ITR (1000 µg mL^−1^)	50 ± 0.005	15 ± 0.008	2 ± 0.001

The mean ± standard deviation of three replicates according to ANOVA (*p* < 0.05).

## Data Availability

The data that support the findings of this study are available on request from the corresponding author.

## References

[1] Lima P.G., Oliveira J.T.A., Amaral J.L., Freitas C.D.T., Souza P.F.N. (2021). Synthetic antimicrobial peptides: Characteristics, design, and potential as alternative molecules to overcome microbial resistance. Life Sci..

[2] Kumar A., Alam A., Rani M., Ehtesham N.Z., Hasnain S.E. (2017). Biofilms: Survival and defense strategy for pathogens. Int. J. Med. Microbiol..

[3] Kovács R., Majoros L. (2020). Fungal quorum-sensing molecules: A review of their antifungal effect against *Candida* biofilms. J. Fungi.

[4] Zarnowski R., Westler W.M., Lacmbouh G.A., Marita J.M., Bothe J.R., Bernhardt J., Lounes-Hadj Sahraoui A., Fontaine J., Sanchez H., Hatfield R.D. (2014). Novel entries in a fungal biofilm matrix encyclopedia. MBio.

[5] Fox E.P., Nobile C.J. (2012). A sticky situation. Transcription.

[6] Kullberg B.J., Oude Lashof A.M.L. (2002). Epidemiology of opportunistic invasive mycoses. Eur. J. Med. Res..

[7] Weig M. (1998). Clinical aspects and pathogenesis of *Candida* Infection. Trends Microbiol..

[8] Baillie G.S. (2000). Matrix polymers of *Candida* biofilms and their possible role in biofilm resistance to antifungal agents. J. Antimicrob. Chemother..

[9] Sasso M., Roger C., Sasso M., Poujol H., Barbar S., Lefrant J.-Y., Lachaud L. (2017). Changes in the distribution of colonising and infecting *Candida* Spp. isolates, antifungal drug consumption and susceptibility in a french intensive care unit: A 10-year study. Mycoses.

[10] LaFleur M.D., Kumamoto C.A., Lewis K. (2006). *Candida albicans* biofilms produce antifungal-tolerant persister cells. Antimicrob. Agents Chemother..

[11] Ramage G. (2002). Investigation of multidrug efflux pumps in relation to fluconazole resistance in *Candida albicans* biofilms. J. Antimicrob. Chemother..

[12] Arendrup M.C., Patterson T.F. (2017). Multidrug-resistant *Candida*: Epidemiology, molecular mechanisms, and treatment. J. Infect. Dis..

[13] Katiyar S., Pfaller M., Edlind T. (2006). *Candida albicans* and *Candida glabrata* clinical isolates exhibiting reduced echinocandin susceptibility. Antimicrob. Agents Chemother..

[14] Souza P.F.N., Marques L.S.M., Oliveira J.T.A., Lima P.G., Dias L.P., Neto N.A.S., Lopes F.E.S., Sousa J.S., Silva A.F.B., Caneiro R.F. (2020). Synthetic antimicrobial peptides: From choice of the best sequences to action mechanisms. Biochimie.

[15] Lima P.G., Souza P.F.N., Freitas C.D.T., Oliveira J.T.A., Dias L.P., Neto J.X.S., Vasconcelos I.M., Lopes J.L.S., Sousa D.O.B. (2020). Anticandidal activity of synthetic peptides: Mechanism of action revealed by scanning electron and fluorescence microscopies and synergism effect with nystatin. J. Pept. Sci..

[16] Oliveira J.T.A., Souza P.F.N., Vasconcelos I.M., Dias L.P., Martins T.F., Van Tilburg M.F., Guedes M.I.F., Sousa D.O.B. (2019). *Mo*-CBP_3_-PepI, *Mo*-CBP_3_-PepII, and *Mo*-CBP_3_-PepIII are synthetic antimicrobial peptides active against human pathogens by stimulating ROS generation and increasing plasma membrane permeability. Biochimie.

[17] Mason A.J., Moussaoui W., Abdelrahman T., Boukhari A., Bertani P., Marquette A., Shooshtarizaheh P., Moulay G., Boehm N., Guerold B. (2009). Structural determinants of antimicrobial and antiplasmodial activity and selectivity in histidine-rich amphipathic cationic peptides. J. Biol. Chem..

[18] Mason A.J., Gasnier C., Kichler A., Prévost G., Aunis D., Metz-Boutigue M.H., Bechinger B. (2006). Enhanced membrane disruption and antibiotic action against pathogenic bacteria by designed histidine-rich peptides at acidic PH. Antimicrob. Agents Chemother..

[19] Galdiero E., de Alteriis E., De Natale A., D’Alterio A., Siciliano A., Guida M., Lombardi L., Falanga A., Galdiero S. (2020). Eradication of *Candida albicans* persister cell biofilm by the membranotropic peptide GH625. Sci. Rep..

[20] Belmadani A., Semlali A., Rouabhia M. (2018). Dermaseptin-S1 decreases *Candida albicans* growth, biofilm formation and the expression of hyphal wall protein 1 and aspartic protease genes. J. Appl. Microbiol..

[21] Sierra J.M., Fusté E., Rabanal F., Vinuesa T., Viñas M. (2017). An overview of antimicrobial peptides and the latest advances in their development. Expert Opin. Biol. Ther..

[22] Seyedjavadi S.S., Khani S., Eslamifar A., Ajdary S., Goudarzi M., Halabian R., Akbari R., Zare-Zardini H., Imani Fooladi A.A., Amani J. (2020). The antifungal peptide MCh-AMP1 derived from *Matricaria chamomilla* inhibits *Candida albicans* growth via inducing ROS generation and altering fungal cell membrane permeability. Front. Microbiol..

[23] Souza P.F.N., Lima P.G., Freitas C.D.T., Sousa D.O.B., Neto N.A.S., Dias L.P., Vasconcelos I.M., Freitas L.B.N., Silva R.G.G., Sousa J.S. (2020). Antidermatophytic activity of synthetic peptides: Action mechanisms and clinical application as adjuvants to enhance the activity and decrease the toxicity of griseofulvin. Mycoses.

[24] Bhuiyan M.S.A., Ito Y., Nakamura A., Tanaka N., Fujita K., Fukui H., Takegawa K. (1999). Nystatin effects on vacuolar function in *Saccharomyce*. Biosci. Biotechnol. Biochem..

[25] Borgers M., Van de Ven M.-A. (1989). Mode of action of itraconazole: Morphological aspects. Mycoses.

[26] Huang Y., Huang J., Chen Y. (2010). Alpha-helical cationic antimicrobial peptides: Relationships of structure and function. Protein Cell.

[27] Benavent C., García-Herrero V., Torrado C., Torrado-Santiago S. (2019). Nystatin antifungal micellar systems on endotracheal tubes: Development, characterization and in vitro evaluation. Pharmazie.

[28] Lang B., Liu S., McGinity J.W., Williams R.O. (2016). Effect of hydrophilic additives on the dissolution and pharmacokinetic properties of itraconazole-enteric polymer hot-melt extruded amorphous solid dispersions. Drug Dev. Ind. Pharm..

[29] Dias L.P., Souza P.F.N., Oliveira J.T.A., Vasconcelos I.M., Araújo N.M.S., Tilburg M.F.V., Guedes M.I.F., Carneiro R.F., Lopes J.L.S., Sousa D.O.B. (2020). RcAlb-PepII, a synthetic small peptide bioinspired in the 2S albumin from the seed cake of *Ricinus communis*, is a potent antimicrobial agent against *Klebsiella pneumoniae* and *Candida parapsilosis*. Biochim. Biophys. Acta-Biomembr..

[30] Staniszewska M., Bondaryk M., Swoboda-Kopec E., Siennicka K., Sygitowicz G., Kurzatkowski W. (2013). *Candida albicans* morphologies revealed by scanning electron microscopy analysis. Braz. J. Microbiol..

[31] Lamiable A., Thévenet P., Rey J., Vavrusa M., Derreumaux P., Tufféry P. (2016). PEP-FOLD3: Faster de novo structure prediction for linear peptides in solution and in complex. Nucleic Acids Res..

[32] Martínez-Rosell G., Giorgino T., De Fabritiis G. (2017). PlayMolecule ProteinPrepare: A web application for protein preparation for molecular dynamics simulations. J. Chem. Inf. Model..

[33] Kim S., Chen J., Cheng T., Gindulyte A., He J., He S., Li Q., Shoemaker B.A., Thiessen P.A., Yu B. (2019). PubChem 2019 update: Improved access to chemical data. Nucleic Acids Res..

[34] Trott O., Olson A.J. (2009). AutoDock Vina: Improving the speed and accuracy of docking with a new scoring function, efficient optimization, and multithreading. J. Comput. Chem..

[35] Morris G.M., Huey R., Lindstrom W., Sanner M.F., Belew R.K., Goodsell D.S., Olson A.J. (2009). AutoDock4 and autodocktools4: Automated docking with selective receptor flexibility. J. Comput. Chem..

